# Self-Relative Evaluation Framework for EEG-Based Biometric Systems

**DOI:** 10.3390/s21062097

**Published:** 2021-03-17

**Authors:** Meriem Romaissa Boubakeur, Guoyin Wang

**Affiliations:** Chongqing Key Laboratory of Computational Intelligence, Chongqing University of Posts and Telecommunications, Chongqing 400065, China; wanggy@cqupt.edu.cn

**Keywords:** electroencephalogram (EEG), biometrics, person identification, identity information, open environment, frequency selection

## Abstract

In recent years, electroencephalogram (EEG) signals have been used as a biometric modality, and EEG-based biometric systems have received increasing attention. However, due to the sensitive nature of EEG signals, the extraction of identity information through processing techniques may lead to some loss in the extracted identity information. This may impact the distinctiveness between subjects in the system. In this context, we propose a new self-relative evaluation framework for EEG-based biometric systems. The proposed framework aims at selecting a more accurate identity information when the biometric system is open to the enrollment of novel subjects. The experiments were conducted on publicly available EEG datasets collected from 108 subjects in a resting state with closed eyes. The results show that the openness condition is useful for selecting more accurate identity information.

## 1. Introduction

Recently, there has been growing interest towards the use of the electroencephalogram (EEG) in biometrics [1]. An EEG-based biometric system allows the automatic recognition of people’s identity using their EEG signals, which are recorded while performing a given task. EEG-based biometric systems have been successfully used in a resting state [2,3,4] and under conditions of visual stimuli [5,6,7], mental tasks [8] and emotional stimuli [9,10]. Many research problems in the EEG-based biometric system have been discussed in the literature, including, but not limited to, channel selection [11], frequency selection [12], EEG permanence [13], task sensibility [14], cryptography [15], feature extraction [16] and EEG classification [17].

The extraction of features from EEG signals is one of the most crucial processing steps in EEG-based biometric systems. It allows the determination of ID vectors containing the extracted identity information from EEG signals for each subject in the system. Indeed, some unique individual-specific features can be extracted from EEG signals due to genetic and environmental factors [18]. Many methods have been used in feature extraction, namely the autoregressive model (AR) [19], power spectral density (PSD) [20], wavelet transform [21], coherence features (COH) [22], shannon entropy [23] and common spatial patterns [24], among others. The AR model was first used in the earlier work of Poulos et al. [25] to predict the ID vectors of four subjects in a resting state, evaluated through the vector quantizer network, and was able to achieve correct classification scores in the range of 72% to 81%. Later, AR features were used in many other works [26,27,28,29]. Furthermore, the authors in [13] showed that AR features demonstrate a discriminative capability that is higher than PSD and COH features in a resting state.

The ID vectors should ensure a clear distinction between subjects of the EEG-based biometric system by acquiring sufficient identity information in these ID vectors. However, the sensitive nature of EEG signals may lead to some loss in the extracted identity information. First, EEG signals are non-stationary signals that reflect all brain activities in general, including cognitive processing, the response to external stimuli and the functioning of the whole body. Therefore, it is hard to comprehensibly extract all identity information from the EEG signals. Second, the noisy characteristics of EEG signals due to their acquisition process might cause some damage in the identity information contained in these signals. Finally, the dispersion of the EEG signals, the limited spatial resolution and the selection of channels might omit some sources containing parts of the identity information. For all these reasons, different quality levels of identity information contained in the ID vectors may be extracted from EEG signals through processing techniques [30]. The selection of a higher quality of identity information is necessary to improve the distinctiveness between subjects in EEG-based biometric systems.

Furthermore, the distinctiveness of EEG signals has been approved with a small number of subjects  [18]. This might be due to the difficulty in setting up the subject for the signal acquisition process. The small number of subjects in [31] has been mentioned as a limitation that is not easy to overcome (23 subjects were used while using wireless low-cost devices to record EEG signals). Additionally, the current EEG-based biometric systems are evaluated in static conditions with a fixed number of subjects. However, in the real world, systems are open to the emergence of novel subjects to be enrolled. Enrollment continuously increases the number of subject classes, and the EEG-based biometric system has to update these additional subject classes.

Nevertheless, the system performance after being updated depends essentially on the quality of identity information extracted from EEG signals. Indeed, we hypothesize that more accurate identity information may ensure better performance stability when increasing the number of subject classes. In this context, the present study proposes a new self-relative evaluation framework for EEG-based biometric systems. The framework aims at the selection of more accurate identity information when the biometric system is open to the enrollment of novel subjects. Alongside the openness condition, the framework simulates the enrollment of a growing number of subjects in steps in order to track any performance degradation after each system update. Moreover, the framework creates different quality levels of identity information extracted from EEG signals through processing techniques. Finally, the openness condition is carried for each level of identity information in order to select the identity information related to the minimal performance degradation observed through openness.

The designed EEG-based biometric system of the current work uses AR features to predict the ID vectors. Moreover, biometrics include two types of application, namely identification and authentication. The designed system aims to use person identification to recognize the identity of any subject from their EEG signals, which are recorded while performing a given task. The conducted analysis uses a freely available EEG dataset [32] containing EEG signals recorded in a resting state with closed eyes from 108 subjects. Therefore, the present study uses the self-relative evaluation framework to evaluate an EEG-based person identification system using AR features extracted from EEG signals in a resting state. This evaluation allows the selection of the identity information that ensures the lowest performance degradation while increasing the number of subjects enrolled in the system.

This paper is structured as follows. Section 2 describes the conceptual representation of the self-relative evaluation framework. Section 3 presents the dataset and the conducted experiments on the self-relative evaluation framework. Section 4 reports the results and discussion. Finally, in Section 5, we provide the conclusion and future perspectives.

## 2. Materials and Methods

### 2.1. Framework Description

**Figure 1:**
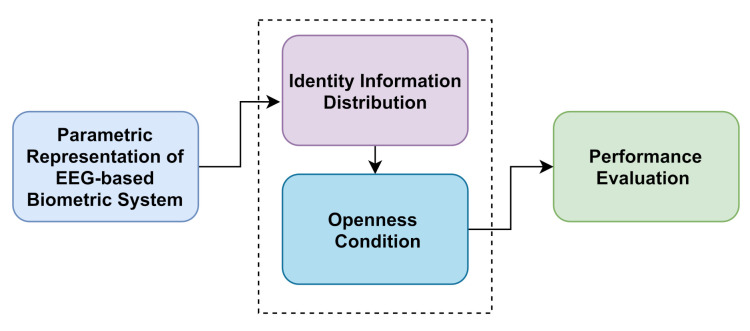
Main parts of the self-relative evaluation framework for an EEG-based biometric system.

This section describes the conceptual representation of the self-relative evaluation framework for EEG-based biometric systems. The proposed framework contains four main parts (see Figure 1):

1. Parametric representation of the system: This presents the parametric representation of the designed EEG-based biometric system through the selected processing techniques.
2. Identity information distribution: This creates different quality levels of identity information contained in EEG signals through processing techniques.
3. Openness condition: This simulates the enrollment of a growing number of labeled subjects through steps. In each step, the system updates the additional subject classes using a class-update strategy.
4. Performance evaluation: This defines the self-relative metrics to measure the performance degradation resulting from the openness condition.

### 2.2. Parametric Representation of the System

**Figure 2:**
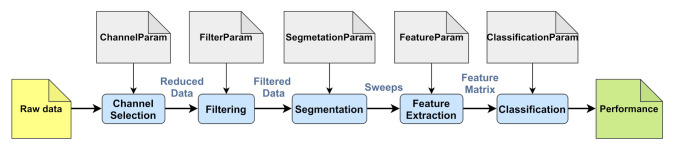
Processing steps of the EEG-based biometric system.

In the EEG-based biometric system, EEG signals are processed through a series of methods, namely channel selection, filtering, segmentation, feature extraction and classification. In each method, input data are processed to provide output data; that is, the input data of the following method (see Figure 2). Besides this, every method includes its necessary input parameters to process the data.

We call a parametric representation of an EEG-based biometric system the set of all input parameters of methods ordered according to the processing sequence in the system. Formally, we note the parametric representation of the system as follows:

(1) $$P=\overset{n}{\underset{i=1}{⋃}}{\left(id,inputParam\right)}_{i}$$

where *n* is the total number of methods used in the processing, id is the method name and inputParam is the set of input parameters of method id.

The EEG-based biometric system presented in our previous work [30] is a person identification system. The current study uses this system along with an update on the filtering step. We briefly present the selected methods to summarize later the parametric representation of the designed system (in Section 3.2.1).

1. Channel selection: selection of a set of *H* channels (an *H*-channel EEG-based person identification system is available).
2. Filtering: Signals are enhanced through two filters:
  - Filter CAR: Common average referencing (CAR) spatial filtering, applied to reduce artifacts related to unsuitable reference choices or unexpected reference variations [33].
  - Low-pass filter: Butterworth filter, used to filter a range of frequency pass bands in EEG signals.
3. Segmentation: Static overlapped segmentation by dividing each EEG signal of duration *V* into segments of duration *v*, with an overlapping factor of α. The total number of segments per class of subject *N* is calculated by $N=\left[\frac{V-v}{v*\left(1-\alpha \right)}\right]+1$.
4. Feature extraction: Burg’s coefficients of an auto-regressive model of order *Q* are used as features. The coefficients extracted from each EEG channel are then concatenated to form a single d-dimensional feature vector d=H*Q to represent the whole segment. The autoregressive model is described by a linear difference equation as follows:
  (2) $$x\left(k\right)=Q+\sum_{i=1}^{Q}a\left(i\right)x\left(t-i\right)+e\left(t\right)$$
  where *Q* is a constant, *Q* stands for the number of parameters of the AR model and $e\left(t\right)$ denotes a white noise input. Burg’s method is used to estimate the AR model parameters.
5. Classification: A multi-classification problem of *E* classes using E*N feature vectors (ID vectors), where *E* is the total number of subjects used in learning and *N* is the total number of feature vectors per subject class. The K-nearest neighbor classifier (KNN) is used to train and test the model through cross-validation. The KNN classifier relies on distance and similarity measures between training and test sets: a test input is assigned to the most common label among the *k* most similar training inputs.

### 2.3. Identity Information Distribution

This section aims to generate different quality levels of identity information extracted from EEG signals through processing techniques (called identity information distributions). Changing the range of some target input parameters of the parametric representation is one possible way to create different quality levels of identity information [30]. In the current study, parameters related to frequency selection are the selected target parameters.

Indeed, EEG signals are composed of several waveforms, each associated with a specific bandwidth. The study of the discriminative properties of each EEG sub-band thus involves the selection of an appropriate sub-band with the most accurate identity information. For this purpose, the Butterworth filter is used to band-pass EEG sub-bands. The target input parameters are both “filter order” and “frequency sub-band”.

Let *P* be the parametric representation of the designed EEG-based biometric system. Let $S_{1}={\left\{o_{i}\right\}}_{i=1}^{m}$ be the range of filter orders and $S_{2}={\left\{f_{j}\right\}}_{j=1}^{r}$ be the range of frequency sub-bands.

The set of identity information distributions $D={\left\{D^{k}\right\}}_{k=1}^{m*r}$ is obtained by processing different filtering assays for the range $S_{1}$ and $S_{2}$, where for each assay the parametric representation *P* is updated with the pair $\langle o_{i},f_{j}\rangle$, resulting in m*r different configurations of the system *P*.

The openness condition is thereafter carried on each $D^{k}\langle o_{i},f_{j}\rangle \;$ in order to measure the self-relative metrics for each identity information distribution.

### 2.4. Openness Condition

#### 2.4.1. Openness Simulation

**Figure 3:**
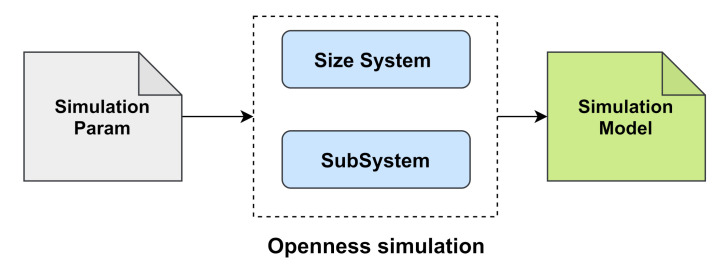
Main components of the openness simulation.

The openness simulation consists of generating—from a fixed number of subjects, labeled from 1 to *E*—several scenarios with an increasing number of subjects in steps. The openness simulation includes two main components (see Figure 3):

- A size system component, which determines the number of subjects included in the system at each step of openness.
  Let *R* be the total number of steps and $T_{n}$ the number of subjects at step *n*, 1≤n≤R. Formally, we note
  (3) $$T_{n}=T_{1}+\sum_{k=2}^{n}Step_{k}\;\;,\;\;T_{n}\leq T_{R}$$
  $Step_{k}$ includes random independent variables with a common distribution *F*. $Step_{k}$ is chosen to simulate a random number of subjects added in each step for a normally increasing number of subjects. Simulation-Param in Figure 3 provides the necessary parameters to generate $Step_{k}$ in addition to the assigned values to $T_{1}$ and $T_{R}$.
- A subsystem component, which determines the set of subject labels included in the system at each step of openness.
  Let $L_{n}$ be the set of subject labels in step *n*, where $size\left(L_{n}\right)=T_{n}$. Moreover, the openness condition means $L_{n-1}\subset L_{n}$ according to Algorithm 1.

The main loop (Lines 2–5) illustrates the gradual enrollment of subject labels in steps to create the subsystem component *L*. At each step *n*, Unknown is the remaining set of subject labels that have not been included in the subsystem component yet. Line 3 randomly selects one possible combination of ($T_{n}-T_{n-1}$) subject labels chosen from the set Unknown to determine Add: the set of subjects’ labels to be enrolled at step *n*. Line 4 defines $L_{n}$: the subject labels of the step *n* by the union of $L_{n-1}$ and Add (the subject labels of the previous step n−1 and the set of enrolled subjects at step *n*). Finally, Line 5 updates Unknown by removing Add: the labels already enrolled in the system.

In order to ensure that results are independent from any given sequence, *M* possible sequences of openness ${\left\{L^{i}\right\}}_{i=1}^{M}$ are generated. Moreover, for the sake of reducing the probability of having two identical sequences, we choose $T_{R}<E$, which gives $M<<<C_{E}^{T_{R}}$ where $C_{E}^{T_{R}}=\frac{E!}{\left(E-T_{R}\right)!T_{R}!}$ is the number of all possible $T_{R}$-combinations chosen from a set of *E* subjects.

Finally, the obtained simulation openness is $S=\left\{T,L\right\}$, where *T* determines the number of subjects to be included in the system in steps and *L* determines the set of subject labels to be included in the system in steps.
**Algorithm 1 Determination of subsystem labels****Input**T: size system component to determinate the number of subjects at each step (R steps).U: label set for all subjects of the dataset labeled from 1 to E. **Output**   L: subsystem component to determinate the set of labels at each step. **START**1: Unknown←U    $T_{0}\leftarrow 0$    $L_{0}\leftarrow \emptyset$ 2: for n=1,…R3:    $Add\leftarrow combination(Unknown,T_{n}-T_{n-1})$4:    $L_{n}\leftarrow L_{n-1}\cup Add$5:    Unknown←Unknown−Add**END**

#### 2.4.2. Class-Update Strategy

**Figure 4:**
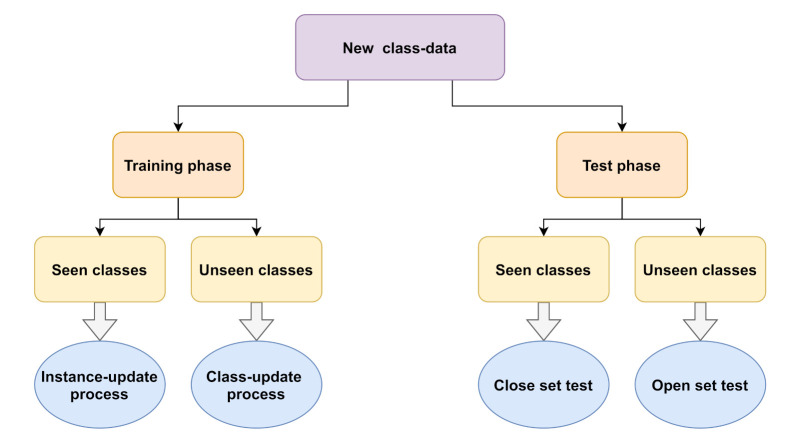
The different possible cases of gradual introduction of class-data in the system.

The gradual enrollment of subjects occurs according to class data in relation to the openness simulation using a given class-update process strategy (see Figure 4). The class-update can be ensured by many learning solutions, including, but not limited to, incremental learning [34], cumulative learning [35] and online learning [36].

In this paper, the complete data are available at each step *j* of openness. Besides this, the number of classes to be learned in each step is small. Therefore, the incremental learning by repetitive batch learning is feasible and sufficient [37].

The openness condition of an openness simulation *S* using repetitive batch learning for the class-update strategy is formally presented as follows:

Let $D={\left\{x_{i}\in R^{d}\right\}}_{i=1}^{E*N}$ be the d-dimensional feature space related to all subjects available in the dataset, labeled in $Y=\left\{y\in N|1\leq y\leq E\right\}$, where *E* is the total number of subjects and *N* is the number of feature vectors per subject class.

Let $f_{j}$ be the classification model learnt at step *j* of an openness simulation $S=\left\{T,L\right\}$, where $L_{j}$ is the set of subject labels included in the system at step *j* and $T_{j}$ is the number of subjects included in the system at step *j*, 1≤j≤R.

**Figure 5:**
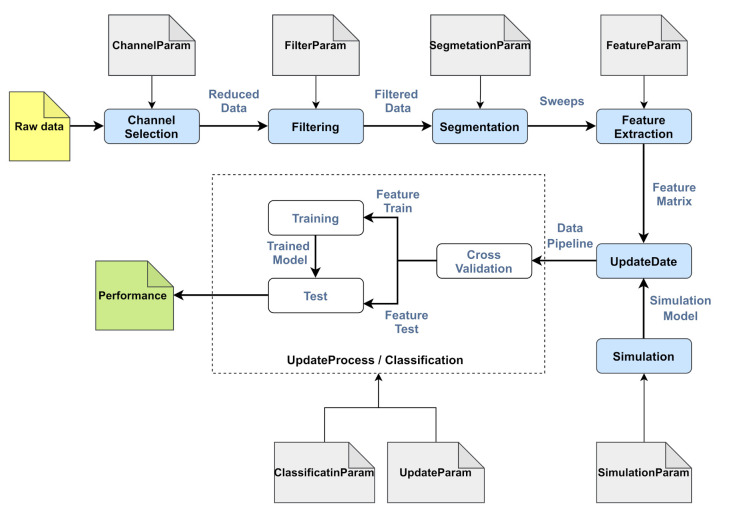
Processing steps of the EEG-based biometric system under the openness condition.

We denote by $D_{j}$ the part of feature space *D* labeled in $L_{j}$ (the update-data mentioned in Figure 5). $D_{j}$ represents the feature space used in learning at step *j*. Formally, we note

(4) $$D_{j}={\left\{x_{i}\in D\;\;|\;\;y_{i}\in L_{j}\right\}}_{i=1}^{T_{j}*N}$$

Through cross-validation (kfold=3), we define the following for every *j*:

- The set of training labeled instances $D^{tr}={\left\{\left(x_{t}^{tr},y_{t}^{tr}\right)\in D_{j}*L_{j}\right\}}_{t=1}^{N_{tr}}$, $x_{t}^{tr}$ is the instance in the feature space, and $y_{t}^{tr}$ is the corresponding subject label.
- The set of testing instances $X^{te}={\left\{x_{t}^{te}\in D_{j}\right\}}_{t=1}^{N_{te}}$, where $x_{t}^{te}$ is a testing instance in the feature space. Besides this, $Y^{te}={\left\{y_{t}^{te}\in L_{j}\right\}}_{t=1}^{N_{te}}$ are the corresponding subject labels for $X^{te}$ to be predicted.
- $D^{tr}$ is used to train the biometric system and learn the classification model $f_{j}$ (repetitive batch learning).
- Through a close set test, $Y^{P}$ is predicted from test instances $X^{te}$ using the learned model $f_{j}$: $Y^{P}=predict\left(f_{j},X^{te}\right)$.
- The classification accuracy $acc_{j}$ is learned using the confusion matrix between $Y^{te}$ and $Y^{P}$.

We note the vector of openness accuracy $oa={\left\{acc_{j}\right\}}_{j=1}^{R}$ representing the accuracy of the system at all steps of openness.

The openness condition is carried on *M* possible sequences of openness ${\left\{L^{i}\right\}}_{i=1}^{M}$ to ensure that the results are independent of any given sequence, resulting in *M* vectors of openness accuracy $OA={\left\{oa_{i}\right\}}_{i=1}^{M}$, where $oa_{i}={\left\{acc_{j}\right\}}_{j=1}^{R}$ is the vector of openness accuracy related to the sequence $L^{i}$.

The mean vector of openness accuracy $OA_{m}={\left\{acc_{m_{j}}\right\}}_{j=1}^{R}$ is defined as follows:

(5) $$OA_{m}=\frac{1}{M}*\sum_{i=1}^{M}oa_{i}$$

The vector $OA_{m}$ is used to describe the system accuracy at each step of openness.

Therefore, the system processing under the openness condition is presented in Figure 5.

The EEG signals from all subjects are processed through the designed EEG-based biometric system. The feature space is then scheduled according to the simulated openness to enroll subjects gradually in steps. The class-update process is then carried out according to the selected model of learning to obtain the mean vector of the openness accuracy $OA_{m}$.

### 2.5. Performance Evaluation

In this section, we define the self-relative metrics under the openness condition for each identity information distribution as described in Algorithm 2.

Line 1 presents the set of identity information distributions $D={\left\{D^{k}\right\}}_{k=1}^{m*r}$ obtained by processing raw EEG signals through the designed EEG-based biometric system *P* for the range of sets of target parameters $S_{1}$ and $S_{2}$. Line 3 indicates that the openness condition is held for each identity information distribution $D^{k}$ using the openness simulation model *S* and the update strategy for learning update to return the mean vectors of openness accuracy $oa_{m_{k}}$. Finally, Line 4 estimates the self-relative metrics from the obtained mean vectors of openness accuracy.
**Algorithm 2 Self relative evaluation****Input**   data: EEG signals of all subjects available in the dataset.   P: parametric representation of the designed EEG-based biometric system.   $S_{1}={\left\{O_{i}\right\}}_{i=1}^{m}$: range of filter orders.   $S_{2}={\left\{f_{j}\right\}}_{j=1}^{r}$: range of frequency sub-bands.   S: openness simulation model.   update: the class-update strategy. **Output**   $OA_{m}={\left\{oa_{m_{k}}\right\}}_{k=1}^{m*r}$: The mean vector of openness accuracy per each distribution of identity information.   $metric={\left\{m_{k}\right\}}_{k=1}^{m*r}$: The self-relative evaluation metrics.  **START**1: $D={\left\{D^{k}\right\}}_{k=1}^{m*r}\leftarrow$identity-information-distribution $\left(data,P,S_{1},S_{2}\right)$ 2: For k=1, ...m*r3:   $oa_{m_{k}}\leftarrow$ openness-condition $\left(D^{k},S,update\right)$4:   $m_{k}\leftarrow$ performance-evaluation $\left(oa_{m_{k}}\right)$ **END**

The self-relative metrics measure the performance degradation when increasing the number of enrolled subjects (openness condition) for each identity information distribution. Therefore, we define two main metrics for the self-relative evaluation as follows:

Let $OA_{m}={\left\{oa_{m_{k}}\right\}}_{k=1}^{m*r}$ be the mean vectors of openness accuracy for each identity information distribution where $oa_{m_{k}}={\left\{acc_{j}\right\}}_{j=1}^{R}$.

- Local relative loss (LRL): the mean degradation in performance between every two successive steps of the openness condition compared to the moving reference step $acc_{j-1}$.
  The condition of performance degradation for local relative loss is $acc_{j-1}>acc_{j}$.
  (6) $$LRL_{k}=100*mean\left(\frac{acc_{j-1}-acc_{j}}{acc_{j-1}}\right)\;\;,\;\;2\leq j\leq R$$
- Global relative loss (GRL): the mean degradation in performance of each step *j* regarding the first step of the openness condition (fixed reference step).
  The condition of performance degradation for global relative loss is $acc_{1}>acc_{j}$.
  (7) $$GRL_{k}=100*mean\left(\frac{acc_{1}-acc_{j}}{acc_{1}}\right),2\leq j\leq R$$

## 3. Datasets and Experiments

### 3.1. Datasets

The online database PhysioNet BCI (archive.physionet.org/pn4/eegmmidb/ (accessed on 16 March 2021) ) [32] contains EEG signals from 109 healthy volunteers collected using a 64-channel system (BCI2000 System). In total, 108 subjects from the 109 volunteers were chosen in order to have the same signal time length for all subjects.

The baseline condition of a resting state with Closed Eyes (1 min EC resting state) provided in this database was used in the current study. The participants were comfortably seated in a dimly lit room on a reclining chair while recording EEG signals. Therefore, the person identification became a multi-classification problem where every subject-class was represented by 1 min EEG signals in an EC resting state processed through the designed EEG-based person identification system.

Table 1 summarizes the description of the dataset used in the experiments.

### 3.2. Experiments

#### 3.2.1. Parametric Representation of the Designed System

The EEG signals were processed through the designed EEG-based person identification system described in Section 2.2. The parametric representation of the designed EEG-based person identification system is presented in Table 2.

The description of the resulting feature space is presented in Table 3. Therefore, the feature space is $D={\left\{x_{i}\in R^{228}\right\}}_{i=1}^{108*19}$ labeled in $Y=\left\{y\in N|1\leq y\leq 108\right\}$.

#### 3.2.2. Identity Information Distribution

Different sets of feature space were created through several filtering assays where the input parameter range was changed in order to generate different levels of identity information distribution. In this paper, the filter order ranges were $S_{1}=\left\{1;2;3;4;5\right\}$ and frequency sub-band ranges were $S_{2}=\left\{[0.5,4];[4,8];[8,13];[13,30];[30,50];[8,30];[4,30];[0.5,30];[0.5,40]\right\}$. Therefore, 45 sets of different levels of identity information distributions were created $D={\left\{D^{k}\right\}}_{k=1}^{5*9}$ where every $D^{k}={\left\{x_{i}\in R^{228}\right\}}_{i=1}^{108*19}$ was a feature space labeled in $Y=\left\{y\in N|1\leq y\leq 108\right\}$.

#### 3.2.3. Openness Condition

**Figure 7:**
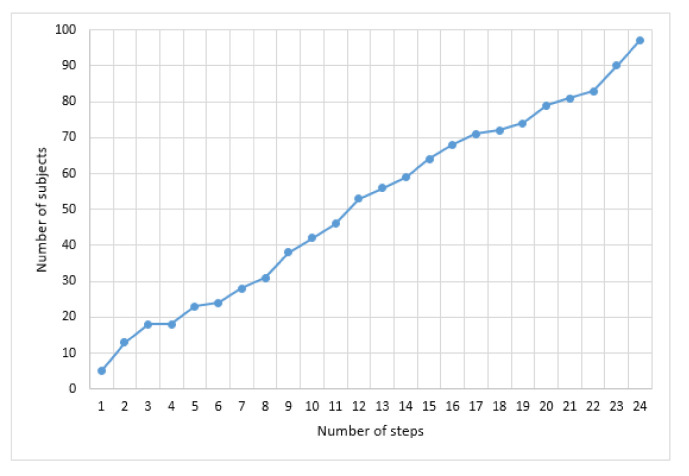
The number of subjects at each step of the openness condition.

The openness simulation $S=\left\{T,L\right\}$ was used, where *T* determined the number of subjects included in the system through steps and *L* determined the set of subject labels included in the system in steps. The growing number of subjects was simulated through the binomial distribution (*n* = 100 and α=0.04), where the number of subjects increased from $T_{1}=5$ to $T_{R}=97$ via R=24 steps (see Figure 7).

**Figure 8:**
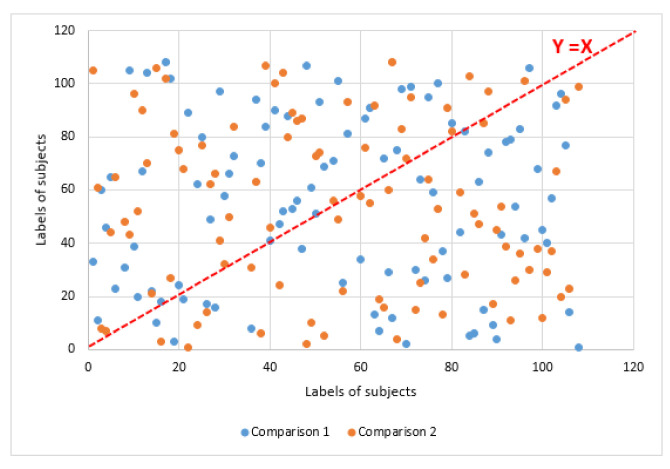
Pairwise comparison between sequences of generated labels.

The sequence of labels *L* was generated as defined in Section 2.4.1 with a total number of subjects of E=108, number of sequences M=10, number of steps R=24 and number of subjects at the last step of $T_{R}=97$. Figure 8 shows a pairwise comparison between three sequences of *L*. The spread of the label distribution refers to the variability of the generated sequences (if sequences were identical, data would be distributed along the line y = x).

Finally, the openness condition was implemented for each identity information distribution $D^{k}$ using the openness simulation model *S*. Through each step *j* of the openness simulation, the person identification was carried out using the class-update strategy described in Section 2.4.2 (repetitive batch learning). The resulting mean vector of openness accuracy was used to describe the mean accuracy of the person identification at each step of the openness simulation.

## 4. Results and Discussion

### 4.1. Framework General Evaluation

**Figure 9:**
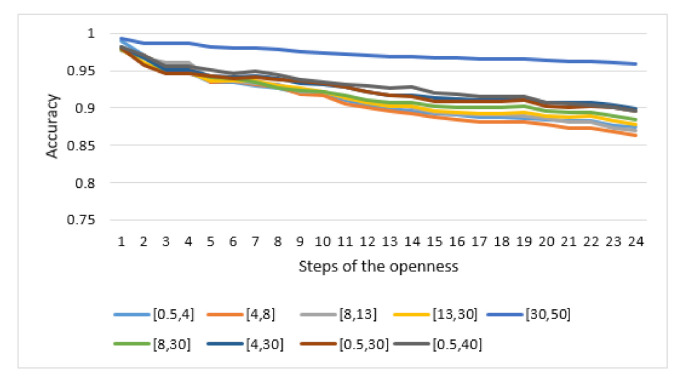
Mean vectors of openness accuracy for each frequency sub-band when the filter order was set to 5 using a 1D digital Butterworth filter.

The openness condition was first implemented with the different identity information distributions generated via the range of frequency sub-bands $S_{2}$ (with a range of one target parameter when the filter order was set to 5). Figure 9 presents the mean vectors of openness accuracy for each frequency sub-band using a 1D digital Butterworth filter. The observed high accuracy indicates that the designed EEG-based person identification was able to accurately distinguish between subjects. However, the results still show a slight degradation of accuracy when the number of subjects was increased in steps. This supports the hypothesis concerning the presence of this degradation and the possibility for it to be used in the self-relative evaluation. Moreover, the velocity of this degradation varies depending on the selected frequency sub-band. On the one hand, this supports the assumption of generating several quality levels of identity information when changing the input parameter range; On the other hand, it supports the hypothesis that more accurate identity information may ensure a better stability of performance when increasing the number of subjects enrolled in the system.

**Figure 10:**
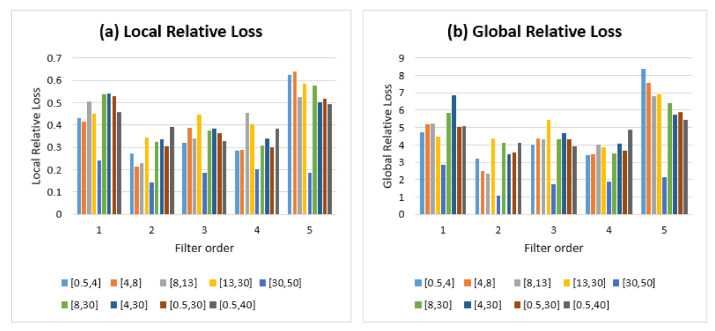
Self-relative metrics: local relative loss (LRL, (**a**)) and global relative loss (GRL, (**b**)) using a 1D digital Butterworth filter.

The openness condition was then implemented for the 45 sets of identity information distributions generated with the range of both filter orders $S_{1}$ and frequency sub-bands $S_{2}$. Figure 10 presents the resulting self-relative metrics using a 1D digital Butterworth filter: local relative loss (Figure 10a) and global relative loss (Figure 10b).

The results confirm the dependency between the amount of identity information and the presence of a degradation in performance under the openness condition, illustrated through both the self-relative metrics of LRL and GRL. Besides, LRL and GRL have almost similar shapes with a difference in scale (from 0.14% to 0.64% for LRL and from 1.07% to 8.35% for GRL). This is explained by the fact that LRL refers to the mean degradation of one step of openness in relation to a prior step (the gap between the number of subjects at both steps is small). Regarding GRL, the mean degradation is between every step of openness in relation to the first step (the gap between the number of subjects at both steps is increasingly wide). This highlights the cumulative effect of openness: the degradation in performance is more important when steps are increasingly spaced out. In the following analysis, the GRL is used to consider the cumulative effect of the openness condition.

### 4.2. Diversity in Identity Information Distributions

This analysis allowed us to measure the power of each target parameter to create diversity in sets of identity information distribution $D^{k}$ observed through ranges of related GRL. Two versions of the Butterworth filter were used to enrich the study, namely a 1D digital filter with the “filter” MATLAB function (filter 1) and zero-phase digital filtering with the “filtfilt” MATLAB function (filter 2).

We define the power of a target parameter by the ratio between the minimum and maximum mean value of the GRL:

(8) $$Power=\frac{Max\;\;value\;\;of\;\;the\;\;GRL}{Min\;\;value\;\;of\;\;the\;\;GRL}$$

Table 4 shows the mean of GRL for each filter order for all combined frequency sub-bands. The results show that the power of the target parameter “filter order” is greater with filter 2 than filter 1 (6.3 for filter 2 compared to 1.9 for filter 1). In this case, the power of the same target parameter depends on the selected filter method (i.e., it is method-dependent).

Table 5 shows the mean of GRL for each frequency sub-band for all combined filter orders. The results show that the power of the target parameter “frequency sub-band” for both filters is equivalent (2.1 for filter 2 compared to 2.6 for filter 1).

Therefore, the input parameters allowed the generation of several quality levels of identity information with diversity. This diversity varied according to the selected parameters and methods.

### 4.3. Openness Condition: Comparison between Static and Self-Relative Evaluations

This analysis shows the effectiveness of using the openness condition in the self-relative evaluation. In this context, we present a comparison between the static evaluation and the self-relative evaluation (GRL). Table 6 shows the comparison between both evaluations for the same designed EEG-based biometric system.

The static evaluation of the designed EEG-based person identification system *P* involved the input target parameters being set to a fixed given pair $\langle o_{i},f_{j}\rangle$ with a fixed number of subjects. This showed that each step of the self-relative evaluation was a reference for a static evaluation and the self-relative evaluation was finally a series of *R* static evaluations joined with a class-update strategy. Formally, we note the context of a static evaluation at a given step *n* as follows:

(9) $$Static\;evaluation\;\left(n\right)=\left\{P,\langle o_{i},f_{j}\rangle ,\langle T_{n},L_{n}\rangle \;|\;\langle o_{i},f_{j}\rangle \in \left(S_{1}*S_{2}\right)\right\}$$

The self-relative evaluation $\left\{P,\left(S_{1},S_{2}\right),\langle T,L\rangle ,update\right\}$ was therefore

(10) $$Self\;Relative\;\;evaluation\;={\left\{Static\;evaluation(n)\;,\;update\right\}}_{n=1}^{R}$$

In the self-relative evaluation, the system was self-evaluated through the performance degradation of the static evaluations along all steps of openness. This degradation was described by the self-relative metrics including the GRL. Moreover, the criterion performance for a static evaluation was the maximal accuracy of classification, whereas the self-relative evaluation employed the minimal GRL (minimal degradation).

We thus chose the boundary steps of the first and last steps of the openness condition as two references for the static evaluation to allow comparison with the self-relative evaluation of the same designed system *P*.

#### 4.3.1. Static Evaluation of the First Boundary Step

The static evaluation of the first step of the designed system *P* required the input target parameters to be set to a fixed pair $\langle o_{i},f_{j}\rangle$ with a fixed number of subjects $T_{1}=5$:

$static\;evaluation\;\left(1\right)=\left\{P,\langle o_{i},f_{j}\rangle ,\langle T_{1},L_{1}\rangle \;|\;\langle o_{i},f_{j}\rangle \in \left(S_{1}*S_{2}\right)\right\}$. Let $A_{1}={\left\{acc_{1_{k}}\right\}}_{k=1}^{5*9}$ be the accuracy set of the static evaluation of the first boundary step related to the 45 configurations of the designed system *P* when $\langle o_{i},f_{j}\rangle \in \left(S_{1}*S_{2}\right)$.

**Figure 11:**
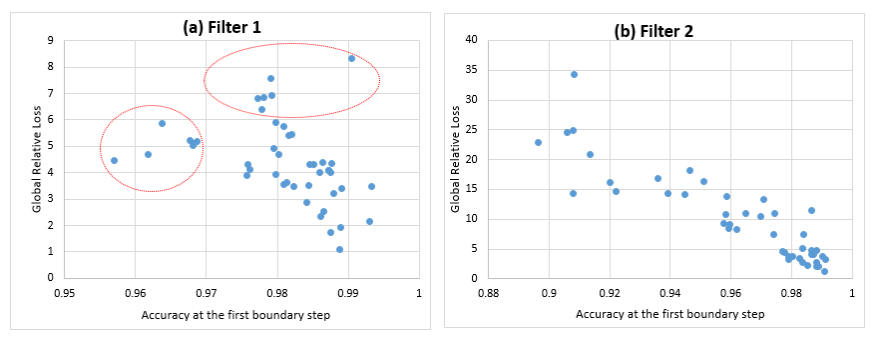
The distribution of the accuracy of the first step against the related GRL.

The GRL regarding the first boundary step represents the future anticipated loss resulting from the openness condition carried from the first boundary step. We plotted the distribution of the first step accuracy $A_{1}$ against the related GRL (see Figure 11). The objective was to determine the supplementary information provided by GRL that may not have been revealed by the accuracy in the first boundary step. For this purpose, we selected the highest accuracies from $A_{1}$ that were significantly equivalent and the set of the related GRLs:

- When the accuracy ∈[0.97,0.999] for filter 1, the related GRL∈[1.07%,8.3%] including 38 configurations of the designed system in the first step.
- When the accuracy ∈[0.96,0.999] for filter 2, the related GRL∈[1.2%,13.2%] including 27 configurations of the designed system in the first step.

The gap between the indicated best accuracies was very small although the gap between the related GRLs was large. This indicates that the openness condition allowed the distinction between the different configurations related to the best accuracies that were significantly equivalent at a given step (static evaluation). This distinction can be made by selecting the configurations associated with the minimal GRL.

Moreover, a better accuracy led to a worse GRL (red circles in Figure 11a). This emphasizes the importance of the self-relative evaluation sight where minimizing the GRL becomes more relevant than the best accuracy at a given fixed step.

#### 4.3.2. Static Evaluation of the Last Boundary Step

The static evaluation of the last step of the designed system *P* allowed the input target parameters to be set to a fixed pair $\langle o_{i},f_{j}\rangle$ with a fixed number of subjects $T_{R}=97$: $static\;evaluation\;\left(R\right)=\left\{P,\langle o_{i},f_{j}\rangle ,\langle T_{R},L_{R}\rangle \;|\;\langle o_{i},f_{j}\rangle \in \left(S_{1}*S_{2}\right)\right\}$. Let $A_{R}={\left\{acc_{R_{k}}\right\}}_{k=1}^{5*9}$ be the accuracy set of the static evaluation of the last boundary step related to the 45 configurations of the designed system *P* when $\langle o_{i},f_{j}\rangle \in \left(S_{1}*S_{2}\right)$.

**Figure 12:**
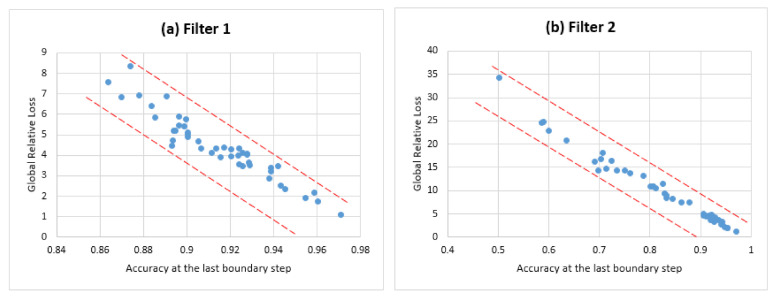
The distribution of last step accuracy against the related GRL.

The GRL regarding the last boundary step represents the past obtained loss resulting from carrying the openness condition to the last boundary step. We plotted the distribution of the last step’s accuracy $A_{R}$ against the related GRL (see Figure 12). The results show that some system configurations result in the same last step accuracy with a gap in the associated GRL (this gap is illustrated by the two asymptotic lines in Figure 12). Therefore, it is important to select the best last step accuracy associated with the minimum GRL.

### 4.4. Framework Decision Making

The decision-making metric ($DMM_{n}=Acc_{n}*\frac{1}{GRL}$) is another possible self-relative metric that can be deduced from the previous results. $DMM_{n}$ combines the static evaluation of a given step *n* with the related GRL. The maximization of $DMM_{n}$ aims at reaching the highest accuracy at step *n* of the openness condition with a minimum GRL.

**Figure 13:**
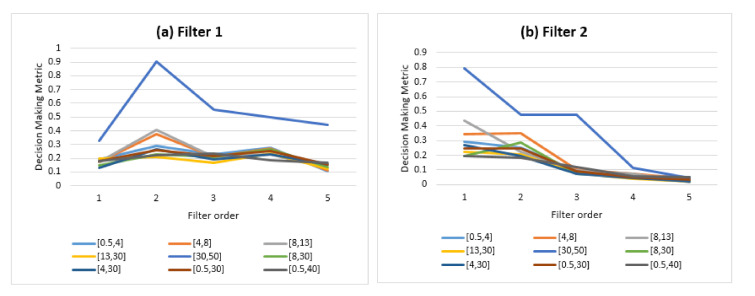
Decision-making metric for the designed EEG-based biometric system.

Figure 13 presents the decision-making metric of the last step $DMM_{R}$ of the designed EEG-based biometric system *P*. The results show that Gamma band [30, 50] outperformed the other sub-bands for both filter 1 and filter 2, making Gamma the most accurate source of identity information compared to the other sub-bands. Furthermore, the power of the target parameter “filter order” to generate a wide diversity of identity information is clearly observed for filter 2 (showing a strong deterioration of identity information when filter order increases).

The highest value of $DMM_{R_{max}}=0.9$ was obtained when filter order was set to 2 and frequency sub-band was set to [30, 50]. Therefore, the best parametric representation of the designed system *P* was for the pair of inputs (2,[30,50]) with a mean GRL of 1.07% under the openness condition (with an increasing number of subjects from $T_{1}=5$ to $T_{R}=97$ via R=24 steps using a repetitive batch learning class-update strategy).

### 4.5. Separability of Classifiers

**Figure 14:**
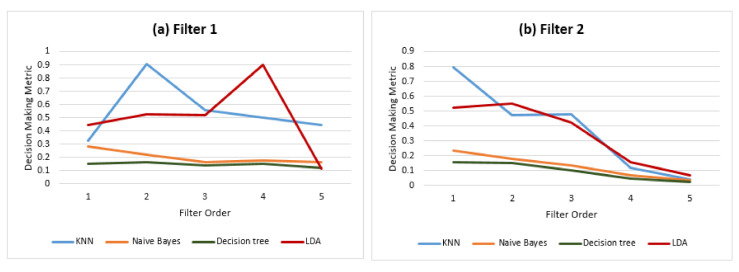
Decision-making metric for the Gamma band and different filter orders for four different classifiers: KNN, Naive Bayes, Decision Tree and Linear Discriminant Analysis (LDA).

In previous results, the Gamma band supported the assumption that more accurate identity information may ensure a better stability of performance when increasing the number of subjects. Figure 14 presents the decision-making metric of the last step using the Gamma band when the filter order was in the range of $S_{1}$ for four different classifiers, K-Nearest Neighbors (KNN), Naive Bayes (NB), Decision Tree (DT) and Linear Discriminant Analysis (LDA). The results show that, with the same amount of identity information (Gamma band), the stability of LDA and KNN outperforms Naive Bayes and Decision Tree under the openness condition. Therefore, the ability of classifiers to set and extend boundaries between classes is another factor that impacts the stability of performance when increasing the number of subjects enrolled in the system.

## 5. Conclusions

In this work, we propose a framework for a self-relative evaluation of an EEG-based biometric system. The framework aims at selecting more accurate identity information when the biometric system is open to the enrollment of novel subjects. Alongside the openness condition, the framework simulates the enrollment of a growing number of subjects through steps in order to track any performance degradation after each system update. Moreover, the framework creates different quality levels of identity information extracted from EEG signals through processing techniques. In this paper, different levels of identity information were created through several assays of frequency selection. Finally, the openness condition was implemented for each level of identity information in order to select the identity information related to the minimal performance degradation observed through openness.

The results presented for the self-relative evaluation of the designed EEG-based biometric system allow us to make the following conclusions.

- Target input parameters of processing techniques allow the generation of several quality levels of identity information with diversity. This diversity varies according to the selected parameters and methods.
- The openness condition evaluated with a fixed reference step (first step) shows that the degradation in performance is more important than moving reference steps, which highlights the commutative effect of openness.
- The openness condition helps to distinguish between different identity information levels that are significantly equivalent at a given fixed step. This distinction is made by selecting the best associated self-relative evaluation.
- The Gamma band outperformed all the other frequency bands, and was thus a potential source of identity information. This result is consistent with previous works [12,38] in which the highest recognition rates were observed in the Gamma band. In this paper, the additional result obtained through the self-relative evaluation was that the Gamma band had the highest ability to carry the openness condition (with an increasing number of subjects from $T_{1}=5$ to $T_{R}=97$ via R=24 steps using a repetitive batch learning class-update strategy) with an optimum GRL of 1.07%.
- The openness condition depends on the ability of the chosen classifier to set and extend boundaries between classes when increasing the number of subjects enrolled in the system.

The self-relative evaluation presents the advantage of selecting more accurate identity information through the openness condition. Besides, the tracking of the system performance alongside the openness condition can be used in the future to carry out predictive studies of system performance for a higher number of subjects. Nevertheless, the openness condition depends on the ability of the chosen classifier to set and extend boundaries between classes when increasing the number of subjects enrolled in the system. Therefore, it is important to properly select the classifier of the designed EEG-based biometric system in order to access more accurate identity information through the openness condition. Moreover, the conducted experiments and the data visualization of the obtained results illustrate one possible use case of the general philosophy of the designed framework. In the future work, many other experiments could be conducted to validate this framework in other contexts and scenarios, including other feature extraction methods (instead of AR features), other mental tasks (instead of resting state with EC), other aspects that influence the extracted identity information such as channel selection and signal segmentation (instead of frequency selection), other distributions in openness simulation (instead of binomial distribution) and other learning models for the class-update strategy (instead of repetitive batch learning) to provide a more incremental and scalable update learning approach.

## Figures and Tables

**Figure 6:**
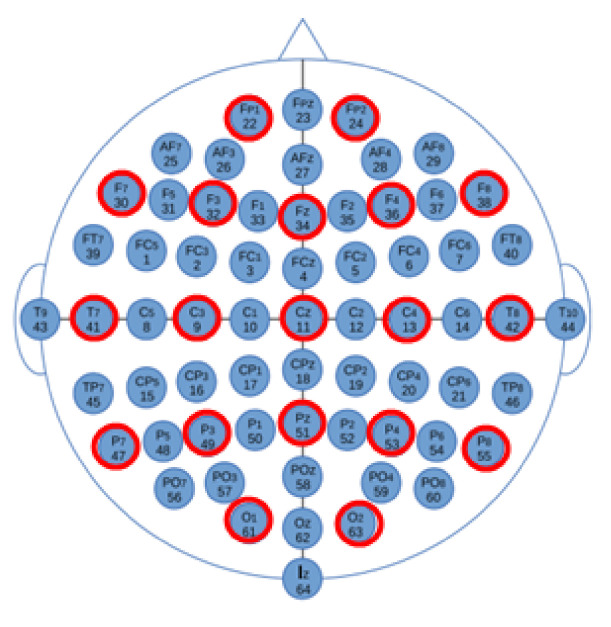
International 10–10 System standards for sensor positioning (the 19 selected channels are shown with red circles).

**Table 1:** Description of the dataset used in the experiments.

Datasets	Information
Mental task	EC
Number of subjects	108
Number of channels	64
Sampling frequency	160 HZ
Signal time length	60 s

**Table 2:** The parametric representation of the designed EEG-based person identification system. CAR: common average referencing; AR: autoregressive; KNN: K-nearest neighbor.

Steps	Method “ID”	Input Parameters
Channel selection	Set selection	- Set of *H* channels (see Figure 6).
Filtering	Filter CAR	- Number of selected channels (H=19).
	Butterworth	- Order of filter *O*.
		- Sampling frequency ($f_{s}=160$ HZ).
		-The selected frequency sub-band *f*.
Segmentation		- Size of segment (v=5).
	Static overlapped	- Overlapping percentage (α=0.4).
	segmentation	- Number of selected channels (H=19).
		- Sampling frequency ($f_{s}=160$ HZ).
Feature Extraction	AR features	- Order of features (Q=12).
		- Coefficients’ estimation (Burg).
Classifier	KNN	- Number of neighbors (k=1).
		- Type of distance (Euclidean).
		- Cross-validation (Kfold = 3).

**Table 3:** Feature space description.

Feature Space Description	Information
Number of classes, E	108
Number of feature vectors per class, N	19
Dimension of feature vector, d	228

**Table 4:** The mean of GRL for each filter order for all combined frequency sub-bands.

Filter Order	1	2	3	4	5	Power
Filter 1	5.03	3.19	4.12	3.65	6.15	1.9
Filter 2	3.34	3.74	8.60	13.12	21.18	6.3

**Table 5:** The mean of GRL for each frequency sub-band for all combined filter orders.

Sub-Bands	[0.5, 4]	[4, 8]	[8, 13]	[13, 30]	[30, 50]	[8, 30]	[4, 30]	[0.5, 30]	[0.5, 40]	Power
Filter 1	4.73	4.63	4.54	5.01	1.94	4.83	4.97	4.49	4.70	2.6
Filter 2	8.70	8.55	11.99	12.12	5.77	11.22	12.10	10.39	9.13	2.1

**Table 6:** Comparison between the static and self-relative evaluations.

Parts	Static Evaluation	Self-Relative Evaluation
System	Parametric representation P	Parametric representation P
Condition	Fixed number of labeled subjects	Openness condition $S=\left\{T,L\right\}$, update
Identity information	Fixed pair of parameters	Parameters range in $S_{1}$ and $S_{2}$
Performance	Maximal accuracy	Minimal GRL
